# 3D imaging and stealth navigation instead of CT guidance for radiofrequency ablation of osteoid osteomas: a series of 52 patients

**DOI:** 10.1186/s12891-019-2963-8

**Published:** 2019-12-01

**Authors:** Ran Ankory, Assaf Kadar, Doron Netzer, Haggai Schermann, Yair Gortzak, Shlomo Dadia, Yehuda Kollander, Ortal Segal

**Affiliations:** 1 Division of Orthopedics, Tel Aviv Sourasky Medical Center affiliated with Tel Aviv University, 153 Arlozorov st app 6, 6492211 Tel Aviv, Israel; 2 Meir Medical Center, Kfar Sava, Israel affiliated with Tel Aviv University, Tel Aviv, Israel; 3 The National Unit for Orthopedic Oncology, Tel Aviv Sourasky Medical Center affiliated with Tel Aviv University, Tel Aviv, Israel

**Keywords:** Osteoid osteoma, O-arm, Radiofrequency ablation, Radiation

## Abstract

**Background:**

Osteoid osteomas are benign bone neoplasms that may cause severe pain and limit function. They are commonly treated by radiofrequency ablation (RFA) through a needle inserted into the nidus of the lesion under CT guidance, which is associated with exposure of young patients to relatively high dose of radiation. The objective of this study was to investigate the amount of radiation, effectiveness and safety of an alternative imaging approach, the 3D image-guided (O-arm) technology and the Stealth navigation.

**Methods:**

We retrospectively reviewed 52 electronic medical files of patients (mean age 24.7 years, range 8–59 years) who were treated with thermal ablation of benign osteoid osteomas guided by the navigated O-arm-assisted technique in our institution between 2015 and 2017. Data were extracted on the associated complications, the reduction in pain at 3 months and one year postoperatively, and the amount of radiation administered during the procedure.

**Results:**

The level of pain on a visual analogue scale decreased from the preoperative average of 7.73 to 0 at the 3-month follow-up. The mean dose-length product was 544.7 mGycm^2^ compared to the reported radiation exposure of 1971–7946 mGycm^2^ of CT-guided radio ablations. The one intra-operative complication was a superficial burn in the subcutaneous lesion in a tibia that was treated locally with no major influence on recovery.

**Conclusions:**

RFA ablation guided by 3D O-arm stealth navigation is as effective as the traditional CT-guided technique with the advantage of lower radiation exposure.

**Trial registration:**

Retrospective study number 0388–17-TLV at Tel Aviv Sourasky Medical Center IRB, approved at 25.10.17.

## Background

Osteoid osteomas are benign bone neoplasms that inflict severe pain. These lesions are small (usually between 10 and 20 mm in diameter) and are located mainly in the diaphysis of long bones. Osteoid osteomas account for 10–12% of all benign bone lesions and mostly affect patients between 5 to 24 years of age [1]. They are a self-limited condition, and are usually resolved without treatment within 1–7 years [1], but many patients require surgical intervention in order to decrease pain and regain function. Until the late 1990’s, the surgical treatment for osteoid osteomas consisted mainly of complete open resection [2]. This treatment was very effective but had several disadvantages. The tumor is difficult to locate and identify under direct vision, thus posing the risks of incomplete resection and tumor recurrence [3]. Additionally, resection of a tumor in a weight-bearing bone might require restrictions of activities or even prophylactic fixation.

The current mainstay of treatment for osteoid osteomas is computerized tomographic (CT)-guided radiofrequency ablation (RFA). This is a minimally invasive procedure with excellent clinical results [4–6]. Complications are rare and may include nerve irritation or superficial burns that are usually resolved without intervention. Rosenthal et al. reported that CT-guided RFA lowered the hospital stay from an average of 4.7 days to 0.18 days [7]. The downside of this procedure is the significant levels of emission of ionizing radiation from the repeated CT scans. High levels of radiation emissions are of special concern when treating osteoid osteomas since they are common among children and young adults and might put them at risk for future radiation-related complications [8].

Several authors have recently described the benefit of using three-dimensional (3D) intra-operative images with an O-arm in combination with a navigation system. They were able to accurately direct implants into demanding anatomical locations without radiation exposure [9–13]. Most of the literature on O-arm-assisted navigation systems is based on spine surgery procedures in which multiple implants (e.g., pedicular screws) are inserted with maximal accuracy and minimal ionizing radiation. The goals of this study were, therefore, to describe a new technique that utilizes a navigation system combined with an O-arm that can help direct the surgeon into bony lesions with minimal radiation to the patient and staff compared to conventional CT-guided procedures. Specifically, we asked: is RFA of osteoid osteomas using 3D image-guided (O-arm) technology and the StealthStation navigation system effective? (2) Does RFA using 3D image-guided (O-arm) technology reduce the amount of radiation exposure compared to CT-guided radio ablations? (3) Is radio frequency ablation using 3D image-guided (O-arm) technology safe?

## Methods

This study was approved by our institutional review board on November 2017 (705–17-TLV). Medical records were reviewed for demographic and clinical data, primary tumor type and anatomical location. Outcome measures were pain scores (utilizing the clinically implemented visual analogue scale) and complications following the procedure. Radiation exposure was quantified by dose-length product (DLP), which is a measurement calculated by the scan length, and the volume CT dose index (CTI). DLP is easily acquired, reproducible, and conforms to regulatory standards [14, 15]. Data were collected using the institutional PACS system by reviewing intraoperative images, dose reports, and interpretative reports.

Between 2015 and 2017, we used the O-arm StealthStation navigation system to treat 52 patients for osteoid osteomas. All 52 patients were available for follow-up for a minimum of 36 months (range, 36–60 months). They included 31 males and 21 females whose mean age at the time of surgery was 24.7 years (range, 8–59 years). None of the patients had received prior surgical treatment for the index lesions. All the patients were diagnosed with osteoid osteomas based on clinical findings and imaging studies. Biopsies of 12 patients were retrieved during the procedure and the specimens were confirmed as osteoid osteomas by a musculoskeletal pathologist. Most of the lesions were located at the femur, while other sites included the tibia, the forearm, and the pelvis (Table 1). Patients with a preoperative pain score of 5 on a visual analogue scale (VAS) were excluded from the study.

**Table 1:** Patient demographic and medical data (*n* = 52)

Parameter	Values
Male sex	31 (60%)
Mean age, years	24.7 (range 8–59)
Lesion location	
Femur	25 (48%)
Tibia	11 (21%)
Radius ulna	5 (9.6%)
Pelvis	4 (7.7%)
Other	7 (13.5%)
Preoperative VAS for pain	7.73 (range 5–10)

*VAS* visual analog scale

**Fig. 1:**
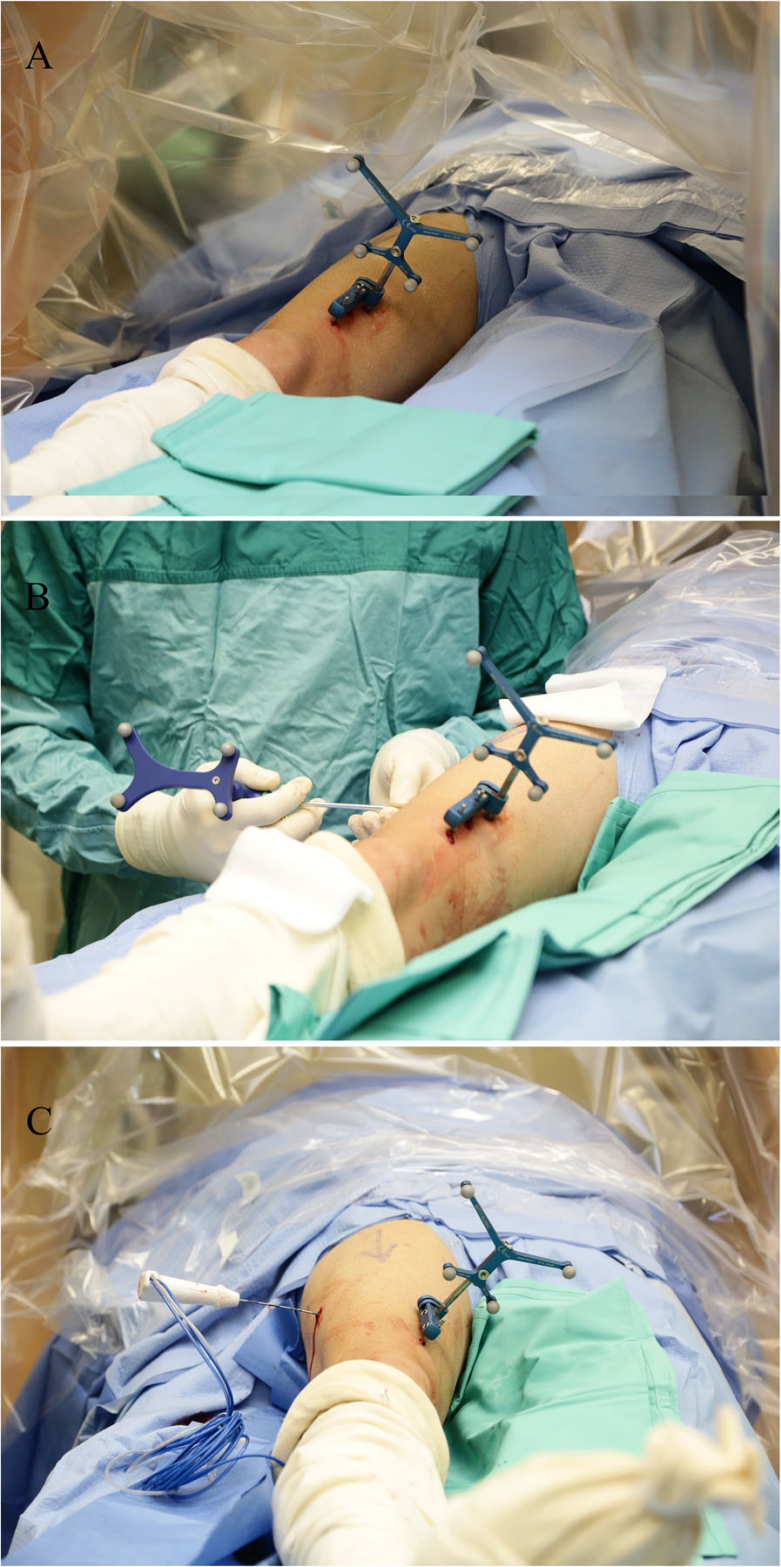
A 20–30 year old patient with right femoral osteoid osteoma treated with radiofrequency ablation guided by three-dimensional (3D) image-guided (O-arm) technology. **a** The Optical tracking frame is fixed to the femur and then calibrated to the navigation system. **b** A navigated Jamshidi needle is introduced to the bone and penetrating the lesion with real time navigation. **c** A Radiofrequency probe needle is inserted into the lesion, followed by ablation according to a protocol of 90 °C for 6–9 min

**Fig. 2:**
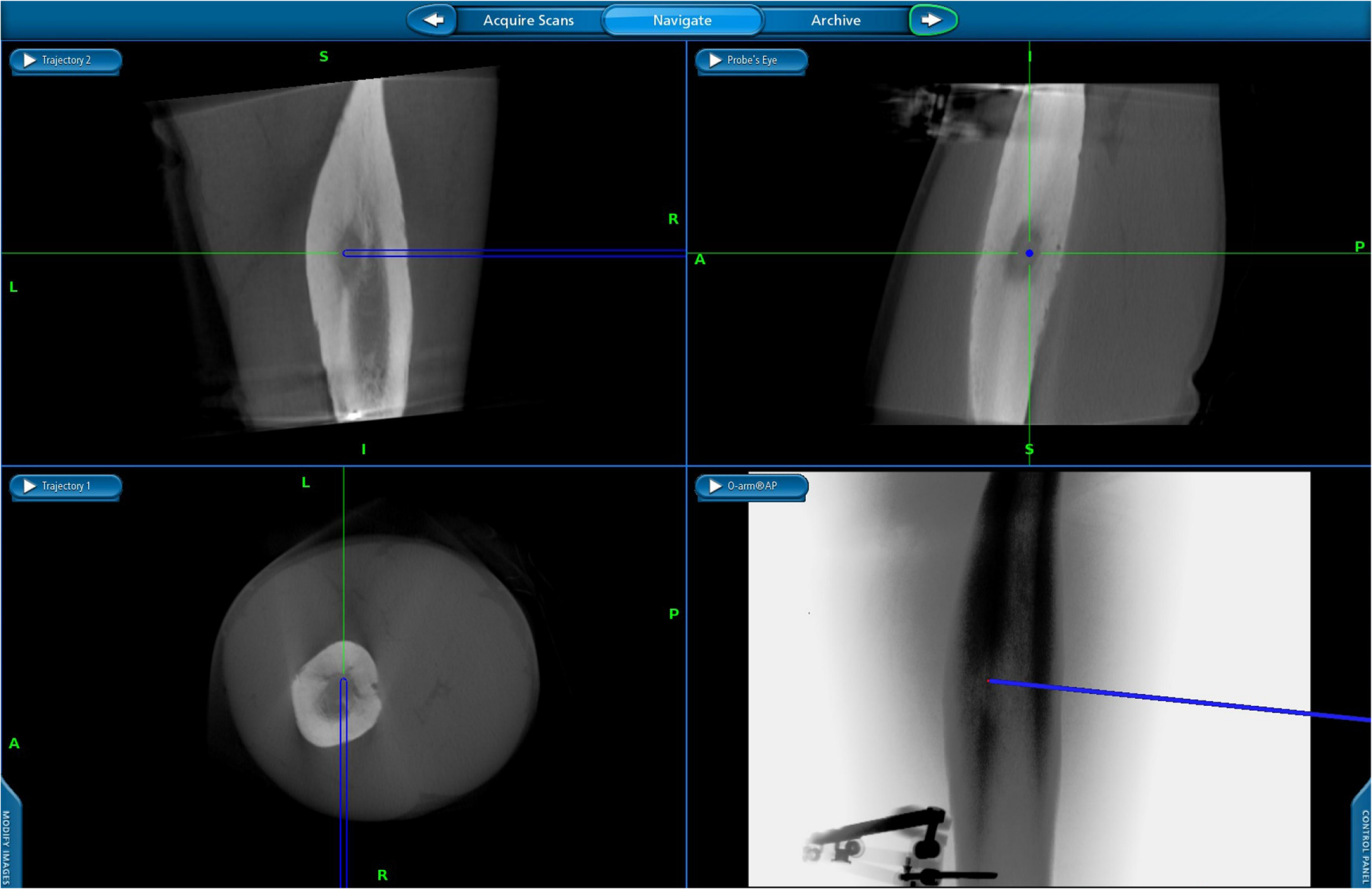
Real time navigation image (O-Arm) of a Jamshidi needle introduced into the osteoid osteoma. The figure presents coronal (upper right panel), sagittal (upper left panel), axial (lower right panel) and 3 dimensional (lower left panel) reconstructions

The surgical procedure was carried out in the operating room with the patient under regional or general anesthesia. The patient was placed on a radiolucent, fluoroscopy-capable operating table, and the affected extremity was prepared and draped in a sterile manner. The optical tracking array was secured to the patient either by silk sutures to the skin in proximity to the lesion or by bony anchor devices, depending on the anatomical location of the lesion. (Fig. 1a). The patient was then scanned by a cone beam CT (O-arm® scanner Medtronic Sofamor, Danek Memphis, TN, USA) to locate the lesion. A 3D image was created by the navigation system (StealthStation®, Medtronic Sofamor, Danek Memphis, TN, USA) and by a set of calibrated navigational instruments (mainly a drill bit or a Jamshidi needle) (Fig. 1b), and the bone and the lesion were penetrated using real-time navigation (Fig. 2). The lesion was then curetted and specimens were sent for biopsy. A 15-cm RF probe needle (RITA Angiodynamics Inc., USA or Covidien Ltd. USA, cool tip 15-cm long) was then advanced without any additional fluoroscopy images or scans. When the needle came in contact with the lesion, a second scan was performed to verify the location of the needle tip, after which the ablation was performed according to a protocol of 90 °C for 6–9 min (Fig. 1c). Lastly, the skin was sutured with 3–0 nylon sutures, and the patient’s skin was examined for burns or other superficial complications. The postoperative protocol included immediate weight bearing.

*Statistical analysis* Means and ranges were used to describe continuous variables, and categorical variables are presented as numbers (percentages). Univariate analyses were performed with the Student’s t-test for continuous variables. The level of significance was set at *p* = 0.05.

## Results

The average duration of the entire procedure was 65 min (range 55–100 min), with an average radio ablation time of 7 min (6–9 min). The navigation system helped place the RF needle accurately into the lesion in all cases, as verified in repeat scans prior to the ablation. All the patients were discharged one day after surgery.

All but one of the 52 patients remained asymptomatic at the 3-week and 3-month follow-up visits. Their pain scores decreased from a preoperative average of 7.73 (range 5–9) to 0.5 (range 0–1) at the first follow-up visit at 3 weeks (*p* = 0.03). Three (5.7%) patients had pain recurrence and underwent repeated ablations within the first year postoperatively. The repeated ablation provided a definitive solution for in all cases. No lesion recurrences were noted at the 36- to 60-month follow-up.

The mean DLP was 544.7 mGycm^2^ (range 180–2959). The variations in DLP were due to the requirement of repeated scans at various anatomical locations in order to ensure the correct placement of the needle tip.

There was one intra-operative complication of a superficial burn in a patient who had a lesion in the tibia. The burn was due to the lesion’s location in a relatively subcutaneous part of the tibia, and it was treated locally without any major sequelae.

## Discussion

The questions we addressed in this retrospective analysis were the effectiveness and safety of the RFA of osteoid osteomas using 3D image-guided (O-arm) technology and StealthSystem navigation for the treatment of osteoid osteomas. Our findings demonstrated that it had provided excellent clinical results in our cohort of 52 patients, with significant pain relief, and mild operative complications. Notably, radiation exposure was reduced compared to the traditional method of inserting the RFA needle under CT guidance.

Osteoid osteomas frequently require surgical treatment for pain relief. While open surgical resection and curettage is an excellent option, it is extensive surgery with the risk of side effects, including a 4.5% fracture rate [2]. Minimal invasive RFA is an acceptable alternative to open surgery. Many studies have shown that RFA provides excellent clinical results with minimal risk and complications [3, 16]. Various imaging modalities have been introduced in recent years in order to guide the RF needle precisely into the lesion’s nidus. The most common modality is standard CT, and the others include ultrasonography, fluoroscopy and magnetic resonance imaging [4, 5, 17]. Lindner at el. presented their results of 58 CT-guided ablations with a 95% success rate after the first ablation attempt and a 100% success rate after a second ablation [17]. Rimondi et al. considered that the key for obtaining a good result is complete removal of the lesion, thus stressing the importance of accurate guidance of the needle into the nidus [18].

3D navigation provides a multi-planar view that allows the surgeon the freedom to find the safest tract to the lesion in real-time navigation without additional radiation exposure. Another advantage of the O-arm approach is the ability to confirm the accurate position of the RF needle. The amount of radiation exposure during traditional CT-guided RFA varies considerably in the literature. Leng et al. reported their experience in 42 ablations and found high levels of radiation exposure (a mean DLP of 7946 ± 3351 mGyCm^2^) [19]. Tsalafoutas et al. reported their experience in 14 RFA procedures and measured a mean DLP of 1971 mGycm^2^ (range 994–3232) [20]. These large variations in radiation exposure can be explained by the different anatomical sites of the lesion and the surgeon’s skill in locating it. The mean DLP of 544.7 mGycm^2^ (range 180–2959 mGycm^2^) in our current report was considerably lower. For convenience, previously reported data regarding DLP of ablations with conventional CT vs O-arm fluoroscopy is summarized in Table 2. Another advantage of the O-arm and stealth navigation is that it is carried out in the operating room, thus enabling a switch to general anesthesia in cases where local anesthesia fails, and as well as transfer to open surgery when required.

**Table 2:** Previously reported data comparing O-arm navigation and CT guidance for osteoid osteoma ablation

Study	Type of guidance	Number of patients	Average ED (mSv)	Average DLP (mGy-cm^2^)
This study (Segal et al)	O-arm with navigation	52	–	544.7
Cheng et al., 2014	O-arm with navigation	23	–	446.6
	Conventional CT guidance	36	–	1058.8
Leng et al., 2011	Conventional CT guidance	42	119.7	7946
Tsalafoutas et al., 2007	Conventional CT guidance	14	35	1976
Perry et al., 2017	Conventional CT guidance	55	39	615

*ED* Effective dose, *DLP* Dose-length product

Our results correlate with those of Cheng at el’s report on the advantages of the O-arm and navigation system. In their comparative study of 66 patients, excellent pain relief was achieved both with conventional CT-guided RFA and with the O-arm navigated RFA. Notably, those authors also noted a significant two-fold reduction in radiation exposure in the O-arm group [21].

Perry et al. have compared use of cone-beam computed tomography (CBCT) with two-axis fluoroscopic navigational overlay and conventional CT guidance for osteoid osteoma ablation in a pediatric cohort. Interestingly, CBCT was associated with more than three-fold reduction in radiation dose (0.12 vs. 0.39 mSv, *p* = 0.02) [22]. These results urge direct comparison between CBCT and O-arm navigation.

Surgical navigation techniques are not without drawbacks. They require an expensive setup and skilled personnel, and intraoperative CT and navigation systems are available in a very limited number of hospitals. The adverse effects of surgical navigations include fractures around the reference frame pin, which was reported in up to 1% of navigated total knee replacements [23].

Several limitations of this study bear mention, It is a retrospective analysis that was carried out on a small group of patients in the absence of a control group. The variation in the levels of radiation is high and could not be accurately compared to radiation exposure in CT-guided RFA without a control group of similar lesion location and number of patients.

## Conclusions

We found that intraoperative O-arm technology in combination with 3D StealthSystem navigation is a safe and effective technique in the treatment of osteoid osteomas and should be considered as an alternative to conventional CT-guided interventions with the aim of reducing radiation exposure, especially among younger patients. Additional research is required to compare O-arm with novel cone-beam CT.

## Data Availability

The datasets used and/or analysed during the current study are available from the corresponding author on reasonable request.
